# Genetic Polymorphisms of the Glycine N-Methyltransferase and Prostate Cancer Risk in the Health Professionals Follow-Up Study

**DOI:** 10.1371/journal.pone.0094683

**Published:** 2014-05-06

**Authors:** Marcelo Chen, Yi-Ling Huang, Yu-Chuen Huang, Irene M. Shui, Edward Giovannucci, Yen-Ching Chen, Yi-Ming Arthur Chen

**Affiliations:** 1 Department of Urology, Mackay Memorial Hospital, Taipei, Taiwan; 2 Department of Cosmetic Applications and Management, Mackay Junior College of Medicine, Nursing and Management, Taipei, Taiwan; 3 School of Medicine, Mackay Medical College, New Taipei City, Taiwan; 4 Department of Public Health, College of Public Health, National Taiwan University, Taipei, Taiwan; 5 Genetics Center, Department of Medical Research, China Medical University Hospital, Taichung, Taiwan; 6 School of Chinese Medicine, China Medical University, Taichung, Taiwan; 7 Department of Epidemiology, School of Public Health, Harvard University, Boston, Massachusetts, United States of America; 8 Department of Nutrition, School of Public Health, Harvard University, Boston, Massachusetts, United States of America; 9 Institute of Epidemiology and Preventive Medicine, College of Public Health, National Taiwan University, Taipei, Taiwan; 10 Research Center for Genes, Environment, and Human Health, College of Public Health, National Taiwan University, Taipei, Taiwan; 11 Department of Microbiology, School of Medicine, Kaohsiung Medical University, Kaohsiung, Taiwan; 12 Center for Infectious Disease and Cancer Research, Kaohsiung Medical University, Kaohsiung, Taiwan; Duke Cancer Institute, United States of America

## Abstract

**Purpose:**

Glycine N-methyltransferase (GNMT) affects genetic stability by regulating the ratio of S-adenosylmethionine to S-adenosylhomocysteine, by binding to folate, and by interacting with environmental carcinogens. In Taiwanese men, GNMT was found to be a tumor susceptibility gene for prostate cancer. However, the association of GNMT with prostate cancer risk in other ethnicities has not been studied. It was recently reported that sarcosine, which is regulated by GNMT, increased markedly in metastatic prostate cancer. We hereby explored the association of GNMT polymorphisms with prostate cancer risk in individuals of European descent from the Health Professionals Follow-up Study (HPFS).

**Methods:**

A total of 661 incident prostate cancer cases and 656 controls were identified from HPFS. The GNMT short tandem repeat polymorphism 1 (STRP1), 4-bp insertion/deletion polymorphisms (INS/DEL) and the single nucleotide polymorphism rs10948059 were genotyped to test for their association with prostate cancer risk.

**Results:**

The rs10948059 T/T genotype was associated with a 1.62-fold increase in prostate cancer risk (95% confidence interval (CI): 1.18, 2.22) when compared with the C/C genotype. The STRP1 ≥16GAs/≥16GAs genotype was associated with decreased risk of prostate cancer when compared with the <16GAs/<16GAs genotype (odds ratio (OR) = 0.68; 95% CI: 0.46, 1.01). INS/DEL was not associated with prostate cancer risk. Haplotypes containing the rs10948059 T allele were significantly associated with increased prostate cancer risk.

**Conclusion:**

In men of European descent, the GNMT rs10948059 and STRP1 were associated with prostate cancer risk. Compared to the study conducted in Taiwanese men, the susceptibility GNMT alleles for prostate cancer had a reverse relationship. This study highlights the differences in allelic frequencies and prostate cancer susceptibility in different ethnicities.

## Introduction

Glycine N-methyltransferase (GNMT, EC2.1.1.20) is a protein with multiple functions. It affects genetic stability by regulating the ratio of S-adenosylmethionine (SAM) to S-adenosylhomocysteine (SAH), by binding to folate [1], [2], and by interacting with carcinogens such as benzo(a)pyrene and aflatoxin B1. We previously showed that these interactions altered the liver detoxification pathway and prevented DNA adduct formation and subsequent cytotoxicity [3]–[5]. In addition, GNMT regulates genes related to detoxification and antioxidation pathways [6]. We previously generated a Gnmt−/− mouse model and showed that Gnmt−/− mice developed chronic hepatitis and glycogen storage disease in the liver [7]. The Gnmt−/− mice were followed till 24 months old and all the female and half of the male mice developed hepatocellular carcinoma (HCC) spontaneously [8]. These findings suggest that GNMT deficiency results in decreased ability in eradicating endogenous free radicals and xenobiotic compounds both at the cellular level and in an animal model; and therefore, homeostasis of GNMT expression is very important for the cellular defense against both endogenous and exogenous stress.

GNMT is expressed in the liver, pancreas, prostate, and kidney [9]. In studies conducted in Taiwanese men, GNMT was found to be a tumor susceptibility gene for HCC [9], [10] and prostate cancer [11]. However, the association of GNMT with prostate cancer in other races or ethnicities remains unclear. A recent study by Sreekumar et al. reported that sarcosine (N-methylglycine) — a differential metabolite regulated by GNMT — increased markedly in tissue and serum of metastatic prostate cancer and was found in the urine after digital rectal examination in organ-confined disease [12]. They showed a stepwise elevation of sarcosine tissue concentration during prostate cancer progression from benign prostate to clinically localized prostate cancer to metastatic disease. GNMT is the enzyme responsible for converting glycine to sarcosine, and they showed that knockdown of GNMT attenuated prostate cancer invasion. However, subsequent studies on the role of sarcosine as a potential biomarker for early prostate cancer detection failed to see any association between sarcosine concentration in the urine and either tumor grade or tumor stage [13], [14], and studies on the association of serum sarcosine levels and prostate cancer reported conflicting results [15]–[17].

The human GNMT gene is located at chromosome 6p12 and we previously reported that it has 3 polymorphic sites in the promoter region that may affect transcriptional activity: short tandem repeat 1 (STRP1), a (GA)n dinucleotide repeat polymorphism, INS/DEL with insertion or deletion of a GAGT tetranucleotide, and rs10948059 [9], [11], [18]. A recent study in Italians by Ianni et al. showed that the GNMT rs9462856 T allele, which is also located in the promoter region upstream of rs10948059, was associated with increased prostate cancer risk [19]. Using the publicly available HapMap version 3, release R2 database, strong linkage disequilibrium was found between Ianni et al.'s rs9462856 and rs10948059 (D′ = 1.000 and r^2^ = 0.760 in Utah residents with Northern and Western European ancestry from the CEPH collection and D′ = 0.946 and r^2^ = 0.737 in Han Chinese in Beijing, China). In this study, we tried to determine the association of the GNMT polymorphisms STRP1, INS/DEL and rs10948059 and prostate cancer risk in Americans of European ancestry.

## Methods

### Study population

In this nested case-control study, incident prostate cancer cases were identified from the ongoing Health Professionals Follow-up Study (HPFS) in the United States. In 1986, 51,529 males in health professions (e.g., dentists, pharmacists, optometrists, osteopath physicians, podiatrists, and veterinarians) were enrolled in HPFS. At baseline, participants completed a questionnaire on demographics, diseases, and health-related topics. These questionnaires were repeated every two years. Information on deaths was obtained from family members, follow-up questionnaires, or a search of the National Death Index and was conducted through March 2011 [20].

Between 1993 and 1995, blood samples were obtained from 18,018 participants, collected in EDTA tubes, shipped by overnight courier, and centrifuged. Aliquots, including plasma, erythrocytes, and buffy coat, were stored in liquid nitrogen, and DNA was extracted using a QIAamp blood extraction kit (Qiagen, Inc., Valencia, CA).

A total of 661 incident prostate cancer cases and 656 controls were identified from the HPFS for our study between 1993 (time of blood return) and January 31, 2000. Matching was one-to-one. Each case was matched with a control that was alive, had not been diagnosed with cancer by the date of the case's diagnosis, and had a prostate specific antigen (PSA) test performed. We restricted the analysis to individuals of European descent to reduce the potential for population stratification.

Demographic data recorded from all subjects included age, body mass index (BMI), and family history of prostate cancer. In prostate cancer cases, clinicopathological data including PSA level, Gleason score and disease stage were recorded. Patients were classified as having aggressive prostate cancer if they had PSA>20 ng/ml, tumor stage ≥III or N1 or M1, or Gleason score ≥8. Lethal prostate cancer cases were those who had metastases at diagnosis or who progressed to metastases or prostate cancer specific death. This study was approved by the Human Subjects Committee at the Harvard School of Public Health and the Human Subjects Committee at Brigham and Women's Hospital. Written consent was given by the patients for their information to be stored in the hospital database and used for research.

### Genotyping of the GNMT genetic polymorphisms

Three polymorphisms of GNMT were analyzed in this study: STRP1, INS/DEL and rs10948059. A TaqMan 5′ nuclease assay was used for genotyping of rs10948059, and automated fragment analysis (GeneScan) was used for genotyping of STRP1 and INS/DEL. Details of the methods and primers used have been described previously [18].

### Statistical analysis

Genotype frequencies were tested for Hardy-Weinberg equilibrium among controls by chi-square test. Odds ratios (OR) and 95% confidence intervals (CI) were computed for the associations between each genotype with prostate cancer by logistic regression models adjusted for age at blood draw. Analyses restricted to prostate cancer subtypes (e.g. aggressive and lethal) used all controls. We used polytomous logistic regression to assess whether the associations were different with aggressive and non-aggressive cancers. The GENECOUNTING software (version 2.0), which implements an estimation-maximization algorithm, was used to estimate the haplotype frequencies and to calculate linkage disequilibrium between the markers [21], [22]. Statistical analyses were done using SAS v9.2 statistical software (SAS Institute, Cary NC), and 2-sided p-value of<0.05 was considered significant.

## Results

Characteristics of the study participants were described in a previous study [23] and selected characteristics are presented in Table 1. In summary, the mean age at blood draw in cases and controls was about 66 years and the mean age at diagnosis in cases was about 69 years. Fourteen percent of prostate cancer cases and 11 percent of controls had a family history of prostate cancer.

**Table 1:** Characteristics of prostate cases (PCa) and controls.

	Cases (n = 661)	Controls (n = 656)
Age at blood draw, mean (sd)	65.8 (7.5)	65.7 (7.4)
Time to PCa diagnosis from blood draw (years), median (IQR)	3.2 (1.7, 4.5)	
Age at diagnosis, mean (sd)	68.9 (7.3)	
<65 years	180(27.2%)	
> = 65 years	481(72.8%)	
Stage, n (%)*		
T1, T2 (N0, M0)	546 (82.6%)	
T3a (N0, M0)	51(7.7%)	
T3b (N0, M0)	24 (3.6%)	
T4 (N0, M0)	0 (0%)	
N1	10 (1.5%)	
M1	10 (1.5%)	
Gleason score, n (%)**		
2 to 6	337 (51.0%)	
7: 3+4 or no major score defined	156 (23.6%)	
7: 4+3	70 (10.6%)	
8 to 10	67 (10.1%)	
PSA at diagnosis, median (IQR)***	7.0 (5.2, 10.8)	
0 to 4	78 (11.8%)	
4.1 to 10	367 (55.5%)	
10.1 to 20	120 (18.2%)	
>20	52 (7.9%)	
Aggressive (PSA at diagnosis>20 or		
Gleason 8–10 or stage T3 or higher)	161 (24.4%)	
Deaths/metastases due to PCa, n (%)	78 (11.8%)	
PCa deaths without recorded metastatic date	48	
Metastases to bone or organ on follow-up	20	
Metastases at diagnosis	10	

* 20 missing data on stage (3.0%).

** 31 missing data on Gleason score (4.7%).

*** 44 missing data on PSA at diagnosis (6.7%).

In prostate cancer cases, the median PSA level was 7.0 (interquartile range: 5.2, 10.8), with the majority of patients (n = 445, 67.3%) having an initial PSA level 10 ng/ml or less. Only 67 (10.1%) cases had a Gleason score between 8 and 10 and the majority (n = 546, 82.6%) of the cases had T1 or T2 disease. Twenty-four percent (161) of the cases were classified as aggressive and 11.8% (78) had distant metastases at diagnosis or progressed to death or metastases.

rs10948059 genotype frequencies in controls were in Hardy-Weinberg equilibrium (p = 0.55). There was an increased risk of total prostate cancer for those with the T/T genotype compared with the C/C genotype (OR = 1.62; 95% CI: 1.18, 2.22) (Table 2). The relationship was suggestively stronger in non-aggressive cases (OR = 1.81; 95%CI: 1.30, 2.53) when compared with aggressive cases (OR = 1.21; 95%CI: 0.76, 1.92) although the p-heterogeneity (0.09) was not statistically significant (Table 3).

**Table 2:** Frequency of GNMT polymorphisms and association with prostate cancer risk.

	Cases n(%)	Controls n(%)	aOR (95% CI)	p-value
**rs10948059** *				
CC	156(25.3)	176(29.0)	1.00 (ref)	
CT	283(45.9)	309(50.8)	1.03 (0.79, 1.35)	0.814
TT	177(28.7)	123(20.2)	1.62 (1.18, 2.22)	0.003
per-allele			1.27(1.08–1.48)	0.003
**STRP1**				
<16GAs/<16GAs	247(38.8)	209(32.8)	1.00 (ref)	
> = 16GAs/<16GAs	332(52.2)	359(56.3)	0.78 (0.61–0.99)	0.039
> = 16GAs/> = 16GAs	57(9.0)	70(11.0)	0.68 (0.46–1.01)	0.058
per-additional GAs			0.81 (0.68–0.97)	0.019
**INS/DEL** **				
DEL/DEL	77(12.3)	86(13.6)	1.00 (ref)	
DEL/INS	415(66.4)	418(66.2)	1.12 (0.80, 1.57)	0.520
INS/INS	133(21.3)	127 (20.1)	1.18 (0.80, 1.75)	0.415
per-additional 4-bp			1.08(0.89–1.31)	0.433

aOR = age-adjusted OR.

*Minor allele frequency in controls = 0.456.

**not in HWE.

**Table 3:** Analysis of polymorphisms according to prostate cancer aggressiveness and lethality.

	Non-aggressive			Aggressive			Lethal		
	cases (%)	aOR (95% CI)	p-value	cases (%)	aOR (95% CI)	p-value	cases (%)	aOR (95% CI)	p-value
**rs10948059** *									
CC	95(22.6)	1.00 (ref)		45(30.4)	1.00 (ref)		20(27.4)	1.00 (ref)	
CT	203(48.2)	1.18 (0.89–1.57)	0.24	64(43.2)	0.80 (0.54–1.18)	0.26	33(45.2)	0.95 (0.53, 1.72)	0.88
TT	123(29.2)	1.81 (1.30–2.53)	0.0005	39(26.4)	1.21 (0.76–1.92)	0.43	20(27.4)	1.41 (0.72, 2.74)	0.32
per-allele		1.36 (1.14–1.63)	0.0006		1.09 (0.84–1.40)	0.52		1.18(0.84–1.67)	0.34
**STRP1**									
<16GAs/<16GAs	168(38.7)	1.00 (ref)		64(40.8)	1.00 (ref)		31(40.3)	1.00 (ref)	
> = 16GAs/<16GAs	227(52.3)	0.78 (0.60–1.01)	0.06	81(51.6)	0.75 (0.52–1.07)	0.11	43(55.8)	0.78 (0.48, 1.29)	
> = 16GAs/> = 16GAs	39(9.0)	0.68 (0.44–1.05)	0.08	12(7.6)	0.57 (0.29–1.12)	0.11	3(3.9)	0.28 (0.08, 0.94)	
per-additional GAs		0.81 (0.67–0.99)	0.04		0.74 (0.55–0.98)	0.04		0.66(0.44–0.98)	0.04
**INS/DEL** **									
DEL/DEL	50(11.6)	1.00 (ref)		19(12.6)	1.00 (ref)		7 (9.6)	1.00 (ref)	
DEL/INS	286(66.5)	1.10 (0.79–1.54)	0.58	103(68.2)	0.92 (0.58–1.46)	0.72	55(75.3)	1.54 (0.67, 3.51)	0.31
INS/INS	94(21.9)	1.17 (0.78–1.74)	0.46	29(19.2)	0.87 (0.49–1.55)	0.64	11(15.1)	1.06 (0.39, 2.86)	0.3
per-additional 4-bp		1.12 (0.91–1.39)	0.29		1.01 (0.74–1.38)	0.97		0.97(0.63–1.50)	0.90

aOR = age-adjusted OR, number of controls = 656.

*MAF in controls = 0.46.

**not in HWE.

p-heterogeneity between aggressive and non-aggressive for rs10948059: CT vs CC = 0.06, TT vs CC = 0.09, per-allele = 0.09.

STRP1 alleles were categorized into two groups: <16GAs and ≥16GAs. Genotypic frequencies in controls were: 32.8% <16GAs/<16GAs, 56.3% <16GAs/≥16GAs, and 11.0% ≥16GAs/≥16GAs. Subjects with ≥16GAs/≥16GAs had decreased risk of prostate cancer when compared to those with <16GAs/<16GAs (OR = 0.68; 95% CI: 0.46, 1.01) (Table 2). This protective association of ≥16GAs was consistently seen in non-aggressive, aggressive and lethal prostate cancers (Table 3).

INS/DEL was not in Hardy-Weinberg equilibrium in controls (p<0.0001). An association between INS/DEL and prostate cancer was not seen (Table 2).

Linkage disequilibrium was not strong among the 3 markers. D′ was 0.837 for STRP1-INS/DEL, 0.634 for INS/DEL-rs10948059, and 0.560 for STRP1-rs10948059. Haplotype analysis of STRP1-rs10948059 showed that, when compared with the other haplotypes, haplotypes with the rs10948059 T allele were significantly associated with increased prostate cancer risk (OR = 1.19, 95%CI: 1.00, 1.42 for 10GAs-T; OR = 1.46, 95%CI: 1.02, 2.10 for 16GAs-T), while those with the rs10948059 C allele tended towards a protective effect against prostate cancer (OR = 0.76, 95%CI: 0.63, 0.92 for 16GAs-C). (Table 4)

**Table 4:** Haplotype frequencies and their association with prostate cancer risk (haplotype STRP1- rs10948059).

Haplotypes*	PCa cases**	Controls**	OR (95% CI)	p-value
10GAs-T	44.1%	39.8%	1.19 (1.00–1.42)	0.048
16GAs-C	25.4%	31.0%	0.76 (0.63–0.92)	0.005
10GAs-C	16.6%	18.7%	0.87 (0.69–1.09)	0.212
16GAs-T	7.5%	5.3%	1.46 (1.02–2.10)	0.039
17GAs-C	4.1%	3.9%	1.05 (0.67–1.63)	0.841

*Only haplotypes with estimated frequencies >1% are listed.

**Estimated numbers of informative haplotypes: PCa cases = 1034, controls = 1003.

## Discussion

In our study of men of European descent, GNMT STRP1 and rs10948059 were indeed associated with prostate cancer risk. Those with an increased number of tandem repeats (≥16GAs/≥16GAs) had a 32% decreased risk of prostate cancer compared to those with less repeats (<16GAs/<16GAs). In addition, those with the rs10948059 T/T genotype had a 62% increased risk of prostate cancer compared to those with the C/C genotype This association appeared to be stronger in non-aggressive compared with aggressive cancers. These findings are in agreement with those of a recent study by Koutros et al., which showed a stronger association between serum sarcosine and non-aggressive prostate cancer, but no association with aggressive prostate cancer [17]. It is therefore possible that GNMT may be a biomarker for early non-aggressive prostate cancer.

Our study results are also supported by a study on the genotypic and phenotypic association of GNMT, which demonstrated that promoters containing either STRP1 10 GAs (<16 GAs) or rs10948059 T allele had significantly higher transcriptional activity than promoters containing STRP1 16 GAs (≥16 GAs) or rs10948059 C allele [18].

Although GNMT acts as a tumor suppressor and was found to be down-regulated in HCC, its role in the pathogenesis of prostate cancer remains unknown. Gnmt−/− mice developed HCC but not prostate cancer, suggesting that other risk factors contributed to the tumorigenesis of prostate cancer besides deficiency or perturbation of the expression level of GNMT. Previously, we used a yeast two-hybrid system to screen proteins interacting with GNMT and found that DEPTOR [24] and NPC2 [25] bound directly with GNMT. We postulate that maybe GNMT exerts its function by interacting with other effectors including DEPTOR and NPC2. DEPTOR is an mTOR inhibitor reported to have an important and more direct role in prostate carcinogenesis [24]. Therefore, further studies on the association of DEPTOR and NPC2 with prostate cancer are needed.

Findings of this study are in contrast to those of our study in Taiwanese men, which showed that the rs10948059 T allele was not significantly associated with non-aggressive prostate cancer (OR = 0.68, 95%CI: 0.36, 1.27) and had a protective association against aggressive prostate cancer (OR = 0.67, 95%CI: 0.47, 0.96) [11]. The distributions of allelic and genotypic frequencies were also significantly different between ethnicities (p<0.0001 for all comparisons). (Table 5) In Taiwanese men, the ≥16 GAs allele was more common (63.8%), while in men of European descent, the <16 GAs allele was more common (61.0%). The <16 GAs allele was not associated with prostate cancer risk in Taiwanese men, while it was associated with a 23 percent increase in prostate cancer risk in men of European descent. In Taiwanese men, the <16GAs/<16GAs genotype was present in 12% of prostate cancer cases and 13% of controls, while in the HPFS, it was present in 39% of prostate cancer cases and 33% of controls. These findings clearly illustrate different allelic and genotypic distributions in Taiwanese and European American men.

**Table 5:** Frequency of the polymorphisms and association of the risk alleles with prostate cancer in Taiwanese prostate cancer study and HPFS controls.

	Taiwanese prostate cancer study^7^	HPFS	p-value*
**rs10948059**			
Allelic frequencies			
C∶T	0.85∶0.15	0.54∶0.46	<0.0001
Genotypic frequencies			
C/C∶C/T∶T/T	0.72∶0.25∶0.02	0.29∶0.51∶0.20	<0.0001
**STRP1**			
Allelic frequencies			
≥16 GAs∶<16 GAs	0.64∶0.36	0.39∶0.61	<0.0001
Genotypic frequencies			
≥16GAs/≥16GAs∶≥16GAs/<16 GAs∶<16GAs/<16GAs	0.41∶0.46∶0.13	0.11∶0.56∶0.33	<0.0001
**INS/DEL**			
Allelic frequencies			
DEL∶INS	0.65∶0.35	0.47∶0.53	<0.0001
Genotypic frequencies			
DEL/DEL∶INS/DEL∶INS/INS	0.42∶0.46∶0.12	0.14∶0.66∶0.20	<0.0001

	OR (95%CI)	OR (95%CI)	
rs10948059 (per-additional T allele)	0.72 (0.52–0.99)	1.27 (1.08–1.48)	
STRP1 (per-reduced GAs)	1.09 (0.88–1.37)	1.23 (1.03–1.47)	
INS/DEL (per-additional 4-bp)	0.89 (0.71–1.12)	1.08 (0.89–1.31)	

*Comparison of *GNMT* allelic and genotypic distributions between Taiwanese population and HPFS (chi-square test).

In Taiwanese men, the rs10948059 C allele was significantly more common than the T allele (85% vs. 15%), while in men of European descent, the C allele was slightly more common than the T allele (54% vs. 46%). In Taiwanese men, the rs10948059 T allele had a protective association against prostate cancer (OR = 0.72). In contrast, in men of European descent, the T allele was associated with increased prostate cancer risk (OR = 1.27). Therefore, the rs10948059 T allele has opposite associations in different ethnic groups. This difference further suggests that it is necessary to validate in a specific ethnicity any associations seen in other ethnicities. Racial and ethnic variations in cancer risk may reflect differences in environmental exposure or differences in susceptibility and biologic response [26]. Polymorphic expression of genes may affect, either by activation or detoxification, the metabolism of carcinogens, such as polycyclic aromatic hydrocarbons, aromatic amines, heterocyclic amines, and other factors. In turn, exposure to different environmental factors may affect the genes and select against specific genetic polymorphisms. Over time, these gene-environment interactions may result in the variable effects seen in different races and ethnicities. It is possible that genes involved in detoxification pathways may be more susceptible to such influences. Kato et al. reported opposite associations of Cytochrome P450IIE1 polymorphisms with lung cancer risk in European and African Americans [27]. Moreover, our study showed that polymorphisms of GNMT, which also participates in detoxification, have variable associations in men of European descent and Asians.

All of the prostate cancer cases and controls in this study were of European descent. It is therefore uncertain whether these GNMT genetic polymorphisms are associated with prostate cancer risk in other ethnicities such as African Americans. A study comprising of 50% Mexican American, 18% European American, 18% African American, 12% Asian and 1% Arab women reported a rs10948059 T allele frequency of 36.4% [28], which is lower than the 45.8% reported in this study. Studies in other ethnic groups, which could all have varying allelic frequencies, are therefore necessary to clarify these associations.

In our previous study in Taiwanese men comprising of 326 prostate cancer cases and 327 controls [11], the allelic frequencies were comparable to those of another study by our group [29]. The frequency of the T allele remained constant at around 15% in controls after pooling subjects from both studies (>600 controls), suggesting that the allelic frequencies were not affected by sample size.

INS/DEL was excluded from haplotype analysis because it was not in Hardy-Weinberg equilibrium (p<0.0001). Haplotype analysis of STRP1-rs10948059 showed that the most common haplotype was 10GAs-T accounting for 40% of controls and 44% of cases, followed by 16GAs-C, and 10GAs-C. (Table 4) Haplotypes with the rs10948059 T allele had ORs greater than 1, suggesting that presence of the rs10948059 T allele per se increased susceptibility to prostate cancer. In Taiwanese men, linkage disequilibrium among the 3 markers was stronger (D′ was 0.988 for STRP1-INS/DEL, 0.948 for INS/DEL-rs10948059, and 0.945 for STRP1-rs10948059). The 10GAs-INS-T haplotype was associated with decreased prostate cancer risk in Taiwanese men (OR = 0.68, 95%CI = 0.48–0.95).

The strength of this study was that we were able to see the variable associations of GNMT with prostate cancer in different ethnicities. A limitation of this study was the lack of data on GNMT expression levels, so a correlation with genotypes could not be made. Immunohistochemical studies may be performed to further elucidate the association of GNMT with prostate cancer in these men of European descent. Immunohistochemical staining using the GNMT monoclonal antibody 14-1 at 1∶25 dilution was previously performed in prostatic tissues obtained from Taiwanese men and a tissue array of Asian men [11]. GNMT expression tended to be higher in non-cancerous than in prostate cancer and tumor-adjacent tissues; and in the cancer tissues, staining was higher in low stage than high stage cancers. These findings are in contrast to those from Song et al.'s study in 148 Japanese men, which showed that high cytoplasmic GNMT expression was correlated with higher Gleason score, higher pathological stage, and lower disease-free survival [30]. While both studies were performed in Asian men, Song et al. used a polyclonal antibody for immunohistochemical staining, so there could be specificity issues, and results from both studies cannot be directly compared. Longitudinal follow-up studies may help clarify the relationship between GNMT expression and disease progression and aggressivity.

Finally, it is worth noting that a higher proportion of subjects in this study had localized (stage I or II) disease (85%) when compared to our previous Taiwanese study (32%) [11]. The study in Taiwanese men was hospital-based, while the HPFS is composed of health care professionals. Health care professionals are more health-conscious and are more likely to have regular physical examinations than the general population^.^ The use of PSA screening was high in the HPFS.

## Conclusions

In men of European descent, the GNMT rs10948059 and STRP1 were associated with prostate cancer risk. Compared to the study conducted in Taiwanese men, the susceptibility GNMT alleles for prostate cancer had a reverse relationship. This study demonstrated the importance of validating associations in different ethnicities, as the allelic and genotypic frequencies were different, and the resulting associations between markers and prostate cancer also differed. Results from this study suggest that GNMT plays a role in prostatic carcinogenesis, but in view of the conflicting results of recent studies on sarcosine, further studies are needed to elucidate the role of GNMT in prostate cancer aggressivity.
